# The Association Between Metabolic Syndrome and Gastric Cancer in Chinese

**DOI:** 10.3389/fonc.2018.00326

**Published:** 2018-08-23

**Authors:** Fangxuan Li, Hui Du, Shixia Li, Juntian Liu

**Affiliations:** ^1^Department of Cancer Prevention, Tianjin Medical University Cancer Institute and Hospital, National Clinical Research Center for Cancer, Tianjin's Clinical Research Center for Cancer, Key Laboratory of Cancer Prevention and Therapy, Tianjin, China; ^2^Department of Obstetrics and Gynecology, The Second Hospital of Hebei Medical University, Shijiazhuang, China

**Keywords:** BMI, gastric cancer, triglycerides, high-density lipoprotein cholesterol, abdominal obesity, diabetes mellitus

## Abstract

**Background:** Metabolic syndrome (MetS) play a carcinogenic role in variety of cancers and influence the prognosis of cancer patients both systemically and hormonally.

**Methods:** The data of clinicopathologic features and MetS of 808 gastric cancer patients and 1,146 randomly healthy controls were analyzed retrospectively.

**Results:** Higher TG level, lower HDL-C level and higher hypertension frequency were observed in all gastric cancer patients when compared with healthy controls. While, gastric cancer patients had greater waist circumference only in females. Among three definitions of MetS, the MetS identified by the Chinese Diabetes Society (CDS) was associated with the most significant increasing risk of gastric cancer. Comparing all gastric cancer patients with healthy controls, OR of gastric cancer was enhanced by various individual components of the MetS, including higher TG level, lower HDL-C level, hypertension and diabetes; In male subgroup, OR of gastric cancer was enhanced by higher BMI, hypertension and diabetes; In females, OR of gastric cancer was enhanced by lower HDL-C, hypertension and diabetes. MetS was associated with poor differentiated carcinoma, more advanced pathological T, N stage and TNM stage of gastric cancer.

**Conclusion:** The presence of MetS and its components were increased in gastric cancer, especially in gastric cancer patients with poor differentiation and advanced stage, which implies that metabolic disorder may play an important role in the development of gastric cancer.

## Introduction

In the worldwide, gastric cancer is the fifth frequently diagnosed cancer with 952,000 new cases and 720,000 deaths in 2012 (1), which is more common in men and in developing countries (2). In East Asia (involving Japan, China, and Korea), gastric cancer is a noticeable public health issue due to its highest morbidity and mortality. A substantial amount of evidences suggested that *Helicobacter Pylori* infection, dietary factors such as salt-preserved foods and salt *per se*, and smoking are risk factors of gastric cancer (3).

Metabolic Syndrome (MetS), which is defined by the presence of at least three out of the five factors: abdominal obesity, elevated triglycerides (TG), decreasing high-density lipoprotein cholesterol (HDL-C), hypertension and high fasting glucose, is becoming an universal and serious health problem all over the world (4). Several expert groups have developed clinical criteria for the metabolic syndrome, while the most widely accepted clinical criteria were produced by the National Cholesterol Education Program-Third Adult Treatment Panel (NCEP-ATP III) and International Diabetes Federation (IDF) (5). Concentrating on racial difference, Chinese Diabetes Society (CDS) recommended a definition of MetS for the Chinese population (6).

Originally, the focus on MetS was primarily concentrating on its effect on increasing cardiovascular disease and type II diabetes mellitus risk. However, recent studies have shown a carcinogenic role of the MetS in many types of cancers. Recent literatures have reported a positive association between clusters of the MetS components and pancreatic cancer (7), adenomatous polyps (8), colon cancer (9), prostate cancer (10), as well as breast cancer, etc (11). Additionally, accumulating evidences had shown that MetS with its systemic and hormonal effects might affect the prognosis of cancer patients (11, 12).

Most components of MetS have been reported to related to the development of gastric cancer (13–15). Several recent studies have found that hyperglycemia and hypercholesterolemia are risk factors for gastric dysplasia (16). MetS components including elevated fasting glucose (100–125 mg/dl), total cholesterol (≥240 m/dL), and moderately elevated low-density lipoprotein cholesterol(LDL-C) (130–159 mg/dL), were associated with a higher incidence of gastric dysplasia (16). Abdominal obesity has been suggested to contribute to increasing risk of gastric adenocarcinoma (17). Meanwhile, the association between diabetes and gastric cancer has also been observed in previous studies (18). In a large-scale, population-based cohort study from Japan, diabetes was linked to elevating 23 and 61% of gastric cancer risk in males and females respectively (19), while previous studies also have investigated that patients with hypertension history had increased risk of gastric cardia adenocarcinoma (20).

Yet, there were some inconsistent results on the linkage between MetS components and gastric cancer, it was reported that obesity has been associated with an increased risk of gastric cancer occurring in the region around the cardia (21) but a decreased risk of distal gastric cancer (22). A study reported there were the positive correlation between excess body weight and gastric cancer among non-Asians, while the positive correlation was not shown in Asians (17). Whereas, concerning on the relationship between diabetes and cancer, the majority of positive studies have been involved in Asian countries (23, 24), few study has been conducted in non-Asians countries.

Additionally, the published epidemiological studies of MetS and gastric cancer yield conflicting results (14, 25). Lindkvist et al. reported that MetS was associated with an increased risk of gastric adenocarcinoma in females, but not in males (25). While, Lin et al. found that as a whole, MetS was related to gastric adenocarcinoma in both women and men (14). Thus, this study aimed to explore the correlation between composite MetS score/the clusters of the MetS components and gastric cancer.

## Methods

### Study population

This was a retrospective, cross-sectional, case-control study. We retrospectively reviewed the clinicopathologic information of gastric cancer patients. Inclusive criteria of gastric cancer patients included: (1) Consecutive patients diagnosed and treated for gastric cancer from February 2011 to June 2013 in the Tianjin Medical University Cancer Institute and Hospital. (2) Patients with gastric adenocarcinoma diagnosed via histopathologic examination. The clinical and histopathological findings were assessed according to the 8th edition of gastric cancer tumor-node-metastasis (TNM) staging system of the Union of International control Cancer (UICC) (26).

Exclusive criteria of patients in this study included: (1) Patients received chemotherapy or radiotherapy before tissue biopsy; (2) Patients with other chronic diseases or infectious diseases;(3) Patients with a history of malignancy. A total of 808 gastric cancer patients had been enrolled in this study, with the mean age of 60.55 ± 10.28 years, including 596 males and 212 females. Laboratory tests and clinical data from 808 consecutive gastric cancer patients were extracted with routine preoperative serum detection and medical data.

To avoid biases of age and gender in our analysis, we randomly selected controls by systematic sampling from healthy physical examinees from February 2011 to June 2013 in Department of Cancer Prevention Center, Tianjin Medical University Cancer Institute and Hospital. Finally, there were 1146 healthy adults had been enrolled in our research, with the mean age of 59.97 ± 9.73 years, including 836 males and 310 females.

All study participants agreed and signed inform consent forms prior to participation. The baseline characteristics of gastric cancer patients and healthy controls were shown in Table 1.

**Table 1:** Baseline characteristics of gastric cancer patients and population-based controls.

**Characteristic**	**Gastric cancer(*n*)**	**Control (*n*)**	***z***	***P***
Total	808	1,146		
Age (mean ± SD), y	60.55 ± 10.28	59.97 ± 9.73	1.255	0.209
Sex (cases)			0.160	0.716
Male	596	836		
Female	212	310		
Smoking status (cases)			1.906	0.197
Yes	149	184		
No	659	962		
Family cancer history (cases)			0.357	0.562
Yes	71	92		
No	637	1054		
Fasting plasma glucose (mmol/L)	5.59 ± 1.69	5.52 ± 1.07	1.039	0.298
Diabetes history (cases)			0.928	0.347
Yes	102	162		
No	706	984		
BMI, kg/m^2^	25.19 ± 3.52	24.88 ± 3.78	1.747	0.080
WC (mean ± SD), cm				
Male	87.12 ± 9.76	86.09 ± 10.11	1.915	0.055
Female	79.48 ± 10.07	77.69 ± 9.36	2.052	0.040
TG (mmol/L)	1.36 ± 0.82	1.25 ± 0.99	2.678	0.007
HDL-C (mmol/L)				
Male	1.35 ± 0.36	1.70 ± 0.40	17.309	< 0.001
Female	1.45 ± 0.51	1.81 ± 0.35	8.938	< 0.001
Systolic BP(mmHg)	122 ± 15	122 ± 17	1.077	0.281
Diastolic BP(mmHg)	80 ± 10	79 ± 11	1.567	0.117
Hypertension history (cases)			42.661	< 0.001
Yes	212	164		
No	596	982		

### Measurement of individual components of MeTS

Three measurements of height, weight and waist circumference were taken by using standardized methods for anthropometric measurements at the time of interview, with the mean used as the final measurement. Weight and height were measured without shoes with light indoor clothes in all patients and healthy controls. Blood pressure was measured in supine or sitting position. BMI was calculated as weight in kilograms divided by the square of height in meters.

Venous blood was collected after 8–12 h fast, either prior to surgical treatment. Blood was drawn post-interview among controls. A 10-mL blood sample was collected according to a standardized protocol, and it was processed into blood fractions (serum, red blood cells, and buff coat), and transported for storage to a specimen bio-repository at the Tianjin Medical University Cancer Institute and Hospital, Tianjin, China. All lipids were measured by a Beckman Coulter AU5821 auto-analyzer (Beckman Coulter, Inc, CA, USA), and included enzymatic colorimetric tests (total cholesterol and TG) and homogeneous enzymatic colorimetric tests (LDL-C and HDL-C), glycohemoglobin analyzer (fasting glucose).

### Definition of MeTS

We defined MetS according to three kinds of international definitions: NCEP-ATP-III (Asian), IDF (Chinese) (5), and Chinese Diabetes Society(CDS) (6). These three definitions of MetS were shown in Table 2.

**Table 2:** Proportion of metabolic syndrome according to three different criteria.

**Criteria**	**Definition factors**	**Metabolic syndrome definition**	Gastric Cancer (n^*^, %)	Control (n^*^, %)	***x^2^***	***P***
	**Total**		**808**	**1,146**		
NCEPATP III (Asian)	WC	>90cm for male, >80cm for female	185 (22.8)	210 (18.3)	6.140	0.014
	TG	≥1.70 mmol/L	152 (18.8)	203 (17.7)	0.384	0.552
	HDL-C	< 1.0 mmol/L for male, < 1.3 for female	241 (29.8)	161 (10.6)	72.194	< 0.001
	Hypertension	Systolic BP ≥ 130, diastolic BP ≥ 85 mmHg or hypertension history	301 (37.2)	332 (29.0)	14.842	< 0.001
	Fasting blood glucose	≥ 6.1 mmol/L or diabetes history	188 (23.3)	162 (14.1)	26.872	< 0.001
	MetS	Criteria 3 or more of the above	164 (20.3)	152 (13.3)	17.293	< 0.001
IDF (Chinese)	WC	>90cm for male, >80cm for female	185 (22.8)	210 (18.3)	6.140	0.014
	TG	≥1.70 mmol/L	152 (18.8)	203 (17.7)	0.384	0.552
	HDL-C	< 1.0 mmol/L for male, < 1.3 for female	241 (29.8)	161 (10.6)	72.194	< 0.001
	Hypertension	Systolic BP ≥ 130, diastolic BP ≥ 85 mmHg or hypertension history	301 (37.2)	332 (29.0)	14.842	< 0.001
	Fasting blood glucose	≥5.6 mmol/L or diabetes history	212 (26.6)	223 (19.5)	12.282	< 0.001
	MetS	WC necessary and any 2 or the above	202 (25.0)	189 (16.5)	21.430	< 0.001
CDS	BMI	≥ 25.0 kg/m^2^	242 (30.3)	312 (27.2)	2.230	0.140
	TG	≥1.70 mmol/L	152 (18.8)	203 (17.7)	0.384	0.552
	HDL-C	< 0.9 mmol/L for male, < 1.0 for female	134 (16.6)	115 (10.0)	18.280	< 0.001
	Hypertension	Systolic BP ≥ 140, diastolic BP ≥ 90 mmHg or hypertension history	212 (26.2)	165 (14.4)	42.661	< 0.001
	Fasting blood glucose	≥6.1 mmol/L or diabetes history	188 (23.3)	162 (14.1)	26.872	< 0.001
	MetS	Criteria 3 or more of the above	154 (19.1)	110 (9.6)	32.510	< 0.001

* *, n was positive patients number*.

*NECP, national cholesterol education program*.

*ATP-III, adult treatment panel-III*.

*IDF, International diabetes federation*.

*CDS, Chinese diabetes society*.

### Statistical analysis

Continuous data were analyzed by *Z* test. Categorical variables were compared by Fisher's exact test and Chi-square test. Continuous variables were described as Mean ± Standard Deviation (SD). Logistic regression analysis was used to estimated ORs and 95% CIs for developing gastric cancer in association with presence of MetS and individual MetS components. The individual MetS components were modeled as meeting the respective cut-point according to definition. Two-sided *P*-values were considered statistical significance at *P* < 0.05. Statistical analysis was performed by SPSS software 20.0.

## Results

### Baseline characteristics of gastric cancer patients and population-based controls

Table 1 showed the baseline characteristics of gastric cancer patients and healthy controls. The differences between-group for each factor entered in the analysis. Compared with healthy controls, gastric cancer patients had higher TG level (*P* = 0.007), lower HDL-C level (*P* < 0.001), and higher frequency of hypertension (*P* < 0.001). While, gastric cancer patients had greater WC only in female subgroup (*P* = 0.040).

### The proportion of participants having MeTS according to three different definitions

As given in Table 2, we compared the proportion of participants with MetS between gastric cancer and healthy controls according to three different definitions of MetS [ATP-III (Asian), IDF (Chinese), and CDS]. Compared with healthy controls, the patients with gastric cancer had higher positive rate of MetS based on all three definitions (All *P* < 0.01). We compared the proportion of cases with abnormal MetS components between gastric cancer and healthy controls and found that increased TC, decreased HDL-C, increased hypertension and fasting blood glucose were more frequently in gastric cancer group according to all the three definitions.

### Enhanced odds ratios (ORS) of gastric cancer according to MeTS and their components by CDS

MetS is one of aging process, thus age and sex can be confounding factors. Therefore, we conducted age and sex adjusted univariate analysis and multivariate analysis for gastric cancer logistic regression analysis. As shown in Table 3, in the total population, the proportion of MetS identified by CDS guidelines in gastric cancer patients (19.1%) was significantly greater than that in healthy control cases (9.6%), and MetS was associated with increased risk for gastric cancer (multivariable-adjusted OR = 2.535, 95%CI:1.805–3.562). While, the MetS was also correlated with increased risk for gastric cancer both in females and males (in females, multivariable-adjusted OR = 1.514, 95%CI:1.197–2.390; in males, multivariable-adjusted OR = 2.683, 95%CI: 1.798–4.004, respectively). Similarly, the magnitude of the OR increasing was also observed according to ATP-III and IDF (Table 3). Because the most significant OR increasing was observed by CDS guideline, in addition, considering Chinese population enrolled in this study, we defined MetS according to CDS definition in the following study.

**Table 3:** Age-adjusted and multivariable ORs and 95% CIs for OR of gastric cancer according to metabolic syndrome.

**Component**	**Total**			**Male**		**Female**	
	**Age adjusted OR (95% CI)**	**Age and sex-adjusted OR (95% CI)**	^*^Multivariable adjusted OR (95% CI)	**Age adjusted OR(95% CI)**	^*^Multivariable adjusted OR (95% CI)	**Age adjusted OR(95% CI)**	^*^Multivariable adjusted^*^ OR (95% CI)
ATP- III	1.862 (1.358–2.558)	1.323 (1.382–2.635)	1.887 (1.350–2.610)	1.821 (1.353–2.450)	1.702 (1.183–2.659)	1.571 (1.233–2.661)	1.504 (1.097–2.390)
IDF	1.939 (1.440–2.611)	1.978 (1.544–2.534)	2.027 (1.465–2.805)	2.002 (1.395–2.871)	1.934 (1.237–2.342)	1.615 (1.296–2.731)	1.542 (1.152–2.450)
CDS	2.455 (1.763–3.418)	2.494 (1.785–3.486)	2.535 (1.805–3.562)	2.720 (1.845–4.010)	2.683 (1.798–4.004)	1.671 (1.233–2.661)	1.514 (1.197–2.390)
BMI	1.121 (0.870–1.444)	1.121 (0.869–1.446)	1.170 (0.901–1.518)	1.783 (1.348–2.358)	1.969 (1.472–2.663)	1.742 (0.917–1.969)	1.818 (0.734–4.506)
TG	1.444 (1.103–2.057)	1.427 (1.000–2.035)	1.431 (1.003–2.041)	1.253 (0.859–1.956)	1.250 (0.817–1.914)	1.455 (0.948– 2.234)	1.414 (0.916–2.183)
HDL-C	1.641 (1.280–2.104)	1.644 (1.281–2.109)	1.642 (1.281–2.106)	1.261 (0.936–1.698)	1.263 (0.936–1.703)	2.323 (1.414–3.817)	2.527 (1.547–4.127)
Hypertension	2.117 (1.595–2.808)	2.115 (1.604–2.778)	2.099 (1.581–2.787)	1.920 (1.391–2.651)	1.864 (1.347–2.580)	2.926 (1.607–5.326.)	2.750 (1.501–5.037)
Diabetes	1.826 (1.372–2.492)	1.862 (1.393–2.489)	1.830 (1.375–2.436)	2.106 (1.164–3.154)	2.062 (1.410–3.402)	1.797 (1.465-2.367)	1.808 (1.473–2.380)

* *BMI ≥25.0 kg/m2; TG ≥ 1.70mmol/L; HDL-C < 0.9mmol/L for male, < 1.09mmol/L for female; Hypertension: systolic BP ≥140, diastolic BP ≥90 mmHg or hypertension history; Diabetes ≥ 6.1 mmol/L or diabetes history; Multivariable-adjusted model: adjusted for age, smoking status (no, yes); family cancer history (no, yes)*.

In the total population, OR of gastric cancer was enhanced by most of the individual components of the MetS by CDS, including higher TG level (multivariable-adjusted OR = 1.431, 95%CI:1.003–2.041), lower HDL-C level (multivariable-adjusted OR = 1.642, 95%CI:1.281–2.106), hypertension (multivariable-adjusted OR = 2.099, 95%CI:1.581–2.787), and diabetes (multivariable-adjusted OR = 1.830, 95%CI:1.375-2.436).

In males, OR of gastric cancer was enhanced by greater BMI (multivariable-adjusted OR = 1.969, 95%CI: 1.472–2.663), hypertension (multivariable-adjusted OR = 1.864, 95%CI: 1.347–2.580) and diabetes (multivariable-adjusted OR = 2.062, 95%CI: 1.410–3.402).

In females, OR of gastric cancer also was enhanced by lower HDL-C (multivariable-adjusted OR = 2.527, 95%CI: 1.547~4.127), hypertension (multivariable-adjusted OR = 2.750, 95%CI:1.501–5.037) and diabetes (multivariable-adjusted OR = 1.808, 95%CI: 1.473–2.380).

### Comparison of clinicopathological characteristics between with or without MeTS and their components by CDS

Consequently, we compared the clinicopathological characteristics between gastric cancer patients with or without MetS based on definition of CDS in Table 4. Among the 808 patients, we found a higher proportion of MetS in poor differentiated carcinoma (*P* = 0.001) and gastric cancer at more advanced T stage (*P* = 0.001), N stage (*P* = 0.001), TNM stage(*P* < 0.001) respectively. Concentrated on individual components of the MetS in all patients, BMI was more frequently increased in proximal gastric cancer (*P* = 0.035), TG increasing was more frequently found in distal gastric cancer (*P* = 0.008) and gastric cancer of Borrmann type IV (*P* = 0.013). Decreased HDL-C was more frequently found in poor differentiated carcinoma (*P* < 0.001), more advanced T stage (*P* = 0.002) and TNM stage (*P* < 0.001). Hypertension was more frequently found in gastric cancer at advanced T stage (*P* = 0.022) and TNM stage (*P* = 0.017).

**Table 4:** Comparison of clinicopathological characteristics between gastric cancer patients with and without metabolic syndrome by CDS definition.

**Features**	MetS (n^*^, %)	BMI (n^*^, %)	TG (n^*^, %)	HDL-C (n^*^, %)	Hypertension (n^*^, %)	Diabetes (n^*^, %)
**LOCATION**						
Proximal	56 (16.8)	114 (34.1)	48 (14.4)	50 (15.0)	88 (26.3)	86 (25.7)
Distal	98 (20.7)	128 (27.0)	104 (21.9)	84 (17.7)	124 (26.2)	102 (21.5)
*x^2^*	1.940	4.744	7.351	1.072	0.004	1.963
*P*	0.173	0.035	0.008	0.337	1.000	0.196
**BORRMANN TYPE**						
I	34 (21.0)	56 (34.6)	20 (12.3)	24 (14.8)	52 (32.1)	44 (27.2)
II	42 (18.8)	70 (31.2)	44 (19.6)	38 (17.0)	50 (22.3)	54 (24.1)
III	72 (18.6)	106 (27.8)	76 (19.6)	68 (17.5)	102 (26.9)	80 (20.6)
IV	6 (17.6)	8 (23.5)	12 (25.3)	4 (11.8)	8 (23.5)	10 (29.4)
*x^2^*	0.512	3.322	10.737	1.210	2.390	3.707
*p*	0.916	0.345	0.013	0.751	0.495	0.295
**HISTOLOGICAL GRADE(-DIFFERENTIATED)**						
Well	20 (11.0)	63 (34.6)	26 (14.3)	10 (5.5)	48 (26.4)	44 (24.2)
Poor	134 (21.4)	179 (28.6)	126 (20.1)	124 (19.8)	164 (26.2)	144 (23.0)
*x^2^*	9.918	2.437	3.151	20.883	0.002	0.109
*P*	0.001	0.119	0.085	< 0.001	1.000	0.765
**T STAGE**						
T1	10 (6.5)	46 (35.4)	32 (24.6)	14 (10.8)	22 (16.9)	28 (21.5)
T2	22 (14.3)	38 (26.4)	24 (16.7)	14 (9.7)	36 (25.0)	32 (22.2)
T3	22 (14.3)	30 (29.4)	18 (17.6)	26 (25.5)	24 (23.5)	20 (19.6)
T4	100 (64.9)	128 (29.6)	78 (18.7)	80 (18.5)	130 (30.1)	108 (25.0)
*x^2^*	17.321	2.736	3.553	15.096	9.646	1.797
*P*	0.001	0.434	0.314	0.002	0.022	0.616
**N STAGE**						
N0	52 (13.3)	114 (29.2)	80 (20.5)	60 (44.8)	92 (28.0)	39 (20.0)
N1	38 (27.1)	42 (30.0)	28 (20.0)	26 (19.4)	40 (28.6)	16 (22.9)
N2	36 (23.4)	50 (32.5)	20 (13.0)	24 (15.6)	44 (28.6)	22 (28.6)
N3	28 (22.6)	36 (29.0)	24 (19.4)	24 (19.4)	36 (29.0)	17 (27.4)
*x^2^*	17.076	0.611	4.313	1.605	2.741	5.969
*P*	0.001	0.894	0.230	0.658	0.433	0.113
**TNM STAGE**						
I	22 (14.3)	70 (33.1)	52 (22.0)	20 (8.5)	52 (22.0)	50 (21.2)
II	46 (29.9)	58 (23.6)	42 (19.1)	56 (25.5)	50 (22.7)	46 (20.9)
III + IV	86 (55.8)	113 (31.8)	36 (16.5)	58 (16.5)	110 (31.2)	82 (26.1)
*x^2^*	21.579	2.135	2.872	23.735	8.125	2.881
*p*	< 0.001	0.334	0.238	< 0.001	0.017	0.237

* *n was positive patients number*.

*MetS, metabolic syndrome were identified by CDS; BMI ≥25.0 kg/m2; TG ≥ 1.70mmol/L; HDL-C < 0.9 mmol/L for male, < 1.09mmol/L for female; Hypertension: systolic BP ≥140, diastolic BP ≥90 mmHg or hypertension history; Diabetes ≥ 6.1 mmol/L or diabetes history*.

In male patients subgroup, significant higher proportion of MetS were observed in poor differentiated carcinoma (*P* < 0.001) and gastric cancer at more advanced T stage (*P* = 0.016), N stage (*P* = 0.014), TNM stage (*P* = 0.001). BMI was more frequently increased in proximal gastric cancer (*P* = 0.048) and gastric cancer patients of III + IV stage (*P* = 0.049). TG increasing was more frequent in distal gastric cancer (*P* = 0.034) and gastric cancer of Borrmann type IV (*P* < 0.001). Decreased HDL-C was more frequently found in poor differentiated carcinoma (*P* < 0.001), more advanced T stage (*P* < 0.001) and TNM stage (*P* < 0.001). Hypertension was more frequently increased in gastric cancer of Borrmann type IV (*P* = 0.041), and gastric cancer at more advanced T stage (*P* < 0.034) and TNM stage (*P* = 0.022).

In female patients subgroup, statistically higher proportion of MetS was also observed in gastric patients at more advanced T stage (*P* = 0.012), N stage (*P* = 0.031), TNM stage (*P* = 0.008). Increased TG was more frequent in T4 stage (*P* = 0.041). Increased TG (*P* = 0.019) and decreased HDL-C (*P* = 0.020) were more frequently found in advanced T stage gastric cancer (Table 5).

**Table 5:** Comparison of clinicopathological characteristics between gastric cancer patients with and without metabolic syndrome by CDS definition in males and females.

**Features**	**Male**						**Female**					
	MetS (n^*^, %)	**BMI (*n*, %)**	TG (n^*^, %)	**HDL-C (*n*, %)**	Hypertension (n^*^, %)	Diabetes (n^*^, %)	MetS (n^*^, %)	BMI (n^*^, %)	TG (n^*^, %)	HDL-C (n^*^, %)	Hypertension (n^*^, %)	Diabetes (n^*^, %)
**LOCATION**												
Proximal	54 (19.1)	95 (33.7)	42 (14.9)	40 (15.2)	80 (28.4)	76 (27.0)	2 (3.8)	19 (36.5)	6 (11.5)	10 (19.2)	8 (15.4)	10 (19.2)
Distal	78 (24.8)	82 (26.1)	68 (21.7)	48 (15.4)	78 (24.8)	66 (21.0)	20 (12.5)	46 (28.9)	36 (22.5)	36 (22.5)	46 (28.8)	36 (22.5)
*x^2^*	2.791	4.081	4.514	0.143	0.949	2.880	3.160	1.120	2.968	0.247	3.693	0.247
*p*	0.114	0.048	0.034	0.730	0.353	0.102	0.113	0.303	0.109	0.691	0.067	0.701
**BORRMANN TYPE**												
I	30 (31.1)	39 (27.8)	10 (8.2)	12 (9.8)	44 (36.1)	38 (31.1)	4 (10.0)	17 (42.5)	10 (25.0)	12 (30.0)	8 (20.0)	6 (11.5)
II	36 (21.2)	55 (32.4)	30 (17.6)	26 (15.3)	38 (22.4)	38 (21.4)	6 (11.1)	15 (27.8)	14 (25.9)	12 (22.2)	12 (22.2)	16 (29.6)
III	60 (22.0)	75 (26.2)	62 (21.7)	46 (16.1)	70 (24.5)	60 (22.0)	12 (11.8)	31 (30.4)	14 (13.7)	22 (21.6)	32 (31.4)	20 (19.6)
IV	6 (33.3)	2 (11.1)	8 (44.4)	4 (22.2)	6 (33.3)	6 (33.3)	0 (0.0)	6 (37.5)	4 (25.0)	0 (0.0)	2 (12.5)	4 (25.0)
*x^2^*	2.048	5.054	18.657	3.584	8.264	5.980	1.051	2.759	4.598	6.066	4.220	3.421
*p*	0.632	0.168	< 0.001	0.310	0.041	0.113	0.789	0.445	0.204	0.108	0.469	0.331
**HISTOLOGICAL GRADE**												
Well-	18 (11.8)	51 (33.6)	20 (13.2)	6 (3.9)	42 (27.6)	40 (26.3)	2 (6.7)	12 (40.0)	6 (20.0)	4 (13.3)	3 (20.0)	4 (13.3)
Poor-	114 (25.7)	120 (27.0)	90 (20.3)	82 (18.5)	116 (26.1)	102 (23.0)	20 (11.0)	59 (32.4)	36 (19.8)	42 (21.0)	24 (26.4)	42 (23.1)
*x^2^*	12.568	2.357	3.806	18.972	0.132	0.697	0.517	0.665	0.000	1.439	0.551	1.439
*p*	< 0.001	0.146	0.053	< 0.001	0.750	0.404	0.747	0.412	1.000	0.339	0.651	0.339
**T STAGE**												
T1	10 (10.6)	30 (31.9)	28 (29.8)	6 (6.4)	16 (17.0)	18 (19.1)	1 (3.3)	16 (44.4)	4 (11.1)	8 (22.2)	6 (16.7)	10 (27.8)
T2	18 (19.6)	28 (29.8)	16 (17.0)	10 (10.6)	22 (23.4)	22 (23.4)	3 (6.0)	10 (20.0)	8 (16.0)	4 (8.0)	14 (28.0)	10 (20.0)
T3	22 (26.5)	21 (24.4)	18 (20.9)	24 (27.9)	20 (23.3)	20 (23.3)	5 (31.3)	7 (43.8)	0 (0.0)	2 (12.5)	4 (25.0)	0 (0.0)
T4	82 (25.1)	95 (29.5)	48 (14.0)	48 (14.9)	100 (31.1)	82 (25.5)	13 (11.8)	33 (30.0)	30 (27.3)	32 (29.0)	30 (27.3)	26 (23.6)
*x^2^*	10.356	1.316	11.193	18.327	8.693	1.635	11.006	7.120	9.980	9.863	1.808	5.545
*p*	0.016	0.725	0.011	< 0.001	0.034	0.652	0.012	0.068	0.019	0.020	0.909	0.136
**N STAGE**												
N0	46 (16.8)	83 (30.3)	60 (21.9)	38 (13.9)	64 (23.4)	54 (19.7)	6 (5.2)	21 (18.1)	20 (17.2)	22 (19.0)	28 (24.1)	24 (20.7)
N1	34 (31.5)	29 (26.9)	20 (18.5)	18 (16.7)	32 (29.6)	28 (25.9)	4 (12.5)	11 (34.4)	8 (25.0)	8 (25.0)	8 (25.0)	4 (12.5)
N2	32 (25.0)	40 (31.2)	18 (14.1)	20 (15.6)	36 (28.1)	38 (29.7)	4 (15.4)	10 (38.5)	2 (7.7)	4 (15.4)	8 (30.8)	6 (23.1)
N3	20 (23.3)	25 (29.1)	12 (14.0)	22 (14.0)	26 (30.2)	22 (25.6)	8 (21.1)	11 (28.9)	12 (31.6)	12 (31.6)	10 (26.3)	12 (31.6)
*x^2^*	10.686	0.629	4.857	0.606	2.720	5.392	8.891	7.271	6.741	3.509	0.511	3.876
*p*	0.014	0.890	0.175	0.895	0.437	0.145	0.031	0.064	0.081	0.320	0.916	0.275
**TNM STAGE**												
I	20 (12.0)	41 (24.6)	42 (25.3)	8 (4.8)	36 (21.7)	36 (21.7)	2 (2.9)	29 (41.4)	10 (14.3)	12 (17.1)	16 (22.9)	14 (20.0)
II	40 (26.0)	36 (23.4)	28 (18.2)	44 (28.6)	34 (22.2)	30 (19.5)	6 (9.1)	16 (24.2)	14 (21.2)	12 (18.2)	16 (24.2)	16 (24.0)
III + IV	72 (26.1)	91 (33.0)	40 (14.5)	36 (13.0)	88 (28.9)	76 (27.5)	14 (18.4)	22 (28.9)	18 (23.7)	22 (28.9)	22 (28.9)	16 (21.1)
*x^2^*	13.611	5.977	8.057	37.023	7.626	4.114	9.661	5.357	2.144	3.686	0.788	0.389
*p*	0.001	0.049	0.018	< 0.001	0.022	0.128	0.008	0.069	0.232	0.158	0.674	0.832

* *n was positive patients number*.

*MetS, metabolic syndrome were identified by CDS; BMI ≥25.0 kg/m2; TG ≥1.70 mmol/L; HDL-C < 0.9 mmol/L for male, < 1.09 mmol/L for female; Hypertension: systolic BP ≥140, diastolic BP ≥90 mmHg or hypertension history; Diabetes ≥ 6.1 mmol/L or diabetes history*.

## Discussion

The MetS is a cluster of metabolic risk factors, involving obesity, hypertension, insulin resistance, hyperglycemia, and dyslipidemia (4). Recently profiling of metabolic intermediates, including amino acids, acylcarnitines, and fatty acids etc., had found a relationship between the subset of these analytes and several chronic conditions and diseases. As recent studies from several other centrals have shown, there is a linkage between MetS and the prevalence of many malignant diseases, such as colorectal cancer, prostate cancer and pancreatic cancer (10, 27, 28). Positive linkages between pancreatic cancer (29)/breast cancer (12) and elements of MetS are also demonstrated.

Recently the researchers gradually focus on the hypothesis that MetS may be associated with the risk of cancer. To date, however, few studies have addressed this issue about gastric cancer. Moreover, the association between MetS and gastric cancer risk was inconsistently. In Lindkvist et al.'s prospective cohort study, composite MetS score assessed by *z*-score standardization was found to be associated with risk of gastric cancer on the borderline significance only in females, but not in males (25). But in their study, this association seemed to be depending on the association between impaired fast glucose and gastric cancer rather than a general tendency in composite MetS score. However, in Lin's study, it was found that MetS, as an overall condition, was associated with gastric adenocarcinoma in both women and men (14). Kim et al.'s study suggested that patients with more than three risk factors of MetS were more likely to occur gastric cancer (30).

MetS components may promote cancer development by generating reactive oxygen species (ROS), increasing hormone production and availability (including estrogen, insulin-like growth factor (IGF), insulin, and adipokines), and forming an energy rich environment (31). There is a possible explanation that the elements of MetS promote cancer through different mechanisms in an additive or synergistic manner. This imbalance of hormones, the redox system and energy availability promotes cancer cells transformation, angiogenesis, migration and proliferation. These mechanisms have been related to BMI, central obesity, insulin resistance, glucose, and TG/fatty acids (11). In a study from China, preoperative MetS components, especially hyperglycemia, were predictive for significant gastric cancer mortality in patients with radical gastrectomy (13). In our study, we also found the increased OR of male and female gastric cancer patients with MetS. Additionally, the relationships between MetS and gastric cancer with poor differentiated carcinoma and advanced disease stage were observed both in males and females.

Major elements of MetS have also been related individually to the development of gastric cancer. Obesity promotes disruptions in multiple metabolic pathways, such as up-regulated sex steroid hormones, insulin, inflammatory mediators and lower levels of adiponectin. It was reported that abdominal obesity is associated with increased hormone levels, such as IGF and adiponectin, which are known to influence cell division, cell death and healing (32). Recent evidences found an increased prevalence of *Helicobacter Pylori* infection in obesity patients, suggesting another indication for the increased incidence of gastric cancer in obesity population (33, 34). A meta-analyses of cohort studies also supported that overweight and obesity were associated with an increased risk of gastric cancer. But, the positive association between excess body weight and gastric cancer could be found only among non-Asians, but not Asians (17). In our study, we also found the BMI could not increase the OR of gastric cancer for the whole gastric cancer patients or females, and the association between BMI and disease stage of gastric cancer was also on borderline. These non-significant results may attribute to small research sample and need further confirm by studies with larger sample sizes from multi-center.

An increased level of serum cholesterol was significantly associated with many malignances. Six prospective studies have examined the possible association between serum cholesterol levels and the development of cancer, but the results of these studies have been inconsistently (28, 35). In Lindkvist et al.'s study, there was no linkage between gastric carcinoma and TG in quintiles or standardized into a *z*-score. However, a significant association was observed in the trend over quintiles in women and when TG were entered as a continuous variable (25). In Kim's study, higher TG levels and lower HDL-C levels were associated with increased risk of gastric cancer (30). In this study, TG and HDL-C were associated with increased OR of gastric cancer in total population. Especially, decreased HDL-C was related to increased OR of female gastric cancer. TG and HDL-C were also associated with poor differentiated carcinoma and advanced disease stage of gastric cancer.

The association between hypertension and increased risk of gastric cancer is also demonstrated by the previous studies, which suggested that patients with self-reported hypertension history may be at a 2.0-fold increased risk of adenocarcinoma of esophagus and gastric cardia (20). The mechanism is still unclear, but it is plausible that hypertension and malignancy may share some common biochemical pathways. For example, increased production of inositol triphosphate and increased levels of cytosolic calcium, which are likely to be involved in the pathogenesis of hypertension and in the early events of cell proliferation, are activated by endogenous mitogens and oncogenes. In this study, increased OR for gastric cancer could be estimated both in male and female patients with hypertension, however, the association between hypertension and advanced disease stage was found only in males.

Fasting glucose was also associated with the risk of gastric cancer in previous studies significantly, which was an independent risk factor for gastric cancer in other studies (24). Several possible pathophysiological mechanisms for a linkage between hyperglycemia and gastric cancer can be proposed. The common suggestion is that the IGF axis can regulate cell proliferation, differentiation, apoptosis, all of these have been implicated in tumorigenesis (36, 37). Hyperglycemia has also been demonstrated to promote formation of ROS by reducing NADPH and formation of advanced glycation end products (38). ROS in turn can promote DNA damage and cancer development (39). In our study, increased OR of gastric cancer can be estimated both in male and female patients with diabetes. However, the association between diabetes and clinicopathological characteristics of gastric cancer was not observed.

In Stocks et al.'s study, the strong association between glucose and gastric cancer in women but not in men was intriguing and raised a suspicion of a modulatory effect by female sex hormones (27). However, to date, there is no clear evidence for this effect from experimental studies. Two Japanese studies form the same prospective cohort have found that moderately impaired fasting blood glucose and hemoglobin A1C (18, 40), respectively, which are associated with an increased risk of gastric cancer, but only in *Helicobacter Pylori* positive subjects. Hence, it is possible that there is an interaction between glucose and *Helicobacter Pylori* infection. Consequently, *Helicobacter Pylori* infection has to be considered as a possible confounder (40). However, in present, as well as many of the previous studies on diabetes and gastric cancer risk, *Helicobacter Pylori* infection was not able to be adjust for, which maybe a limitation in gastric cancer risk estimation.

In conclusion, the MetS is associated with increased risk of gastric cancer in both men and women. Most of the components of MetS have also been linked individually to the gastric cancer developing. The MetS and its components were associated with poor differentiated and more advanced gastric cancer, which implied that metabolic disorder might play an important role in the development of gastric cancer.

## Ethics statement

Ethics approval and consent to participate: This research project was approved by the Ethics Committee of Tianjin Cancer Institute and Hospital. Written consents were obtained from each patient. Consent for publication: Written consents were obtained from each patient to publishing their pathological images as represent Figures.

## Author contributions

FL and JL analyzed and interpreted the patient data. FL and HD was a major contributor in writing the manuscript. SL and HD was analyzed and interpreted and revised the manuscript. All authors read and approved the final manuscript.

### Conflict of interest statement

The authors declare that the research was conducted in the absence of any commercial or financial relationships that could be construed as a potential conflict of interest.
